# Adjuvant Effect of Bacille Calmette–Guérin on Hepatitis B Vaccine Immunogenicity in the Preterm and Term Newborn

**DOI:** 10.3389/fimmu.2018.00029

**Published:** 2018-01-24

**Authors:** Annette Scheid, Francesco Borriello, Carlo Pietrasanta, Helen Christou, Joann Diray-Arce, Matthew A. Pettengill, Sweta Joshi, Ning Li, Ilana Bergelson, Tobias Kollmann, David J. Dowling, Ofer Levy

**Affiliations:** ^1^Department of Pediatric Newborn Medicine, Brigham and Women’s Hospital, Boston, MA, United States; ^2^Department of Medicine, Division of Infectious Diseases, Boston Children’s Hospital, Boston, MA, United States; ^3^Harvard Medical School, Boston, MA, United States; ^4^Precision Vaccines Program, Division of Infectious Diseases, Boston Children’s Hospital, Boston, MA, United States; ^5^Department of Translational Medical Sciences, Center for Basic and Clinical Immunology Research (CISI), University of Naples Federico II, Naples, Italy; ^6^WAO Center of Excellence, Naples, Italy; ^7^Neonatal Intensive Care Unit, Department of Clinical Sciences and Community Health, Fondazione IRCCS Ca’ Granda Ospedale Maggiore Policlinico, Università degli Studi di Milano, Milan, Italy; ^8^Division of Newborn Medicine, Boston Children’s Hospital, Boston, MA, United States; ^9^Thomas Jefferson University, Philadelphia, PA, United States; ^10^Medical Eli Lilly, Shanghai, China; ^11^Department of Pediatrics, British Columbia Children’s Hospital, Vancouver, BC, Canada

**Keywords:** Bacille Calmette–Guérin, hepatitis B vaccine, preterm, newborn, innate cytokine profiles, HBV-specific antibodies

## Abstract

Immunization is key to protecting term and preterm infants from a heightened risk of infection. However, preterm immunity is distinct from that of the term, limiting its ability to effectively respond to vaccines routinely given at birth, such as hepatitis B vaccine (HBV). As part of the Expanded Program on Immunization, HBV is often given together with the live-attenuated vaccine Bacille Calmette–Guérin (BCG), known to activate multiple pattern-recognition receptors. Of note, some clinical studies suggest BCG can enhance efficacy of other vaccines in term newborns. However, little is known about whether BCG can shape Th-polarizing cytokine responses to HBV nor the age-dependency of such effects, including whether they may extend to the preterm. To characterize the effects of BCG on HBV immunogenicity, we studied individual and combined administration of these vaccines to cord newborn and adult human whole blood and mononuclear cells *in vitro* and to neonatal and adult mice *in vivo*. Compared to either BCG or HBV alone, (BCG + HBV) synergistically enhanced *in vitro* whole blood production of IL-1β, while (BCG + HBV) also promoted production of several cytokines/chemokines in all age groups, age-specific enhancement included IL-12p70 in the preterm and GM-CSF in the preterm and term. In human mononuclear cells, (BCG + HBV) enhanced mRNA expression of several genes including *CSF2*, which contributed to clustering of genes by vaccine treatment *via* principle component analysis. To assess the impact of BCG on HBV immunization, mice of three different age groups were immunized subcutaneously with, BCG, HBV, (BCG + HBV) into the same site; or BCG and HBV injected into separate sites. Whether injected into a separate site or at the same site, co-administration of BCG with HBV significantly enhanced anti-HBV IgG titers in mice immunized on day of life-0 or -7, respectively, but not in adult mice. In summary, our data demonstrate that innate and adaptive vaccine responses of preterm and term newborns are immunologically distinct. Furthermore, BCG or “BCG-like” adjuvants should be further studied as a promising adjuvantation approach to enhance immunogenicity of vaccines to protect these vulnerable populations.

## Introduction

Infectious diseases are a leading cause of childhood death with neonatal infection accounting for ~40% of mortality in those <5 years of age, ~7 million cases, and 700,000 deaths per year (1). Within the neonatal population, prematurity, defined as birth at <37 weeks of gestational age (GA), is the single most important cause of death in the first month of life and the second largest cause of death after pneumonia in children <5 years of age (1). Most preterm births (84%, 12.5 million) occur at >32 weeks of gestation (2). At this GA, many preterm newborns can survive with cost-effective supportive care. The mortality from neonatal infection in preterm infants has increased over the last 20 years (1). Moreover, preterm newborns remain at elevated risk of infection through 18 years of age (3). Accordingly, global progress in child survival and health to 2015 and beyond will depend on optimizing preventative care for preterm and term infants with vaccines one of the most effective biomedical approaches for disease prevention.

Vaccine-mediated prevention of infections is limited by reduced or distinct immune responses in early life (4). The current necessity for repeated vaccine booster doses to obtain full protection leaves a window of susceptibility in both the preterm and term infant during the first 6 months of life (5). Alongside efforts at maternal immunization, enhancement of responses to early-life vaccines *via* use of novel adjuvantation systems that demonstrate age-specific immune-enhancing activity is an attractive approach to address this problem. To date, development of pediatric vaccines has largely relied on *ad hoc* studies of adult vaccines, and has not taken the age-dependent development of the immune system into account (6). This holds true for vaccination of the preterm as well: the Advisory Committee on Immunization Practices (ACIP) of US Centers for Disease Control and Prevention as of 2011 has recommended that, with respect to most vaccines, preterm infants be immunized with the full-recommended dose according to the same schedule as full-term infants. However, even thought vaccine immunogenicity in preterm infants is often distinct compared with term responses (7–10), there remains a research knowledge gap. Specifically, hepatitis B vaccine (HBV) immunization is delayed in the preterm due to empiric evidence of reduced immunogenicity. In preterm newborns <1,500–1,800 g birth weight or <34–35 weeks gestation, a three-dose vaccine series of HBV induces protective antibody (Ab) titers in only ~45–85% of patients as opposed to 90–100% in more mature infants (11).

Consideration of vaccine adjuvantation in early life must take into account that newborn innate and adaptive immune cells exhibit distinct activation profiles in response to pattern-recognition receptor (PRR) agonists. However, activation of some PRRs in newborns, such as toll-like receptors (TLRs) 7/8, can induce an adult-like response (12, 13). For example, the germinal center reaction that drives the magnitude and persistence of the Ab response is impaired early in life but can be enhanced with certain TLR agonists (14, 15). Of note, preterm innate and adaptive immunity is distinct from that of both term newborns and adults. For example, preterm monocytes exhibit attenuated PRR-mediated Th1 and Th17-cytokine responses (16). Herein, we assessed whether a live-attenuated vaccine Bacille Calmette–Guérin (BCG), known to activate multiple PRRs (17, 18), might exert adjuvant activity in the context of neonatal HBV immunization.

BCG is the most commonly administered vaccine worldwide and when administered at birth is safe and effective in reducing the rates of infantile tuberculous (TB), meningitis, and disseminated miliary disease (19). It is the only routinely administered neonatal vaccine that induces a Th1-polarized immune response (20). BCG administration may also, in an age-dependent manner, induce beneficial heterologous (“non-specific” or “trained”) immunity against unrelated pathogens and stimuli (21–25), impact responses to other vaccines (20, 26–28), and immune-modulate in the context of allergic diseases as well (29).

Some limited clinical studies have suggested that BCG may enhance responses to other, vaccines such as Oral Polio Vaccine and HBV (20). We posited that co-administration of BCG with HBV could induce greater innate and adaptive immune responses, including acute cytokine induction and HBV-specific Ab production. Employing *in vitro* human blood and mononuclear cell assays we demonstrated that (BCG + HBV) enhanced cytokine and chemokine production on both the protein and mRNA level. Strikingly, *in vivo* immunization of neonatal [day of life (DOL)-0 and -7] and adult mice demonstrated that combined (BCG + HBV) vaccination induced anti-HBV-specific Ab titers in all three age groups at 21 days post-immunization relative to immunization with HBV alone. Overall, our studies provide fresh insight into a vaccine–vaccine interaction that may be the basis of enhanced immunization strategies for vulnerable preterm and term newborn populations.

## Materials and Methods

### Cord Blood Collection

Moderate to late preterm (28 2/7–34 6/7 weeks GA) and term cord blood was collected at The Brigham and Women’s Hospital and the Beth Israel Deaconess Medical Center, both tertiary care centers for delivery and postnatal care of the preterm and term newborns. The details of our preterm study cohort are outlined in Table 1. The de-identified newborn cord blood (~15–60 ml) was collected immediately after caesarian section or vaginal delivery of the placenta from a larger placental or umbilical vein under sterile conditions, as previously described (30). No cord blood samples from newborns born to human-immunodeficiency virus-positive mothers were included. Samples were collected from both male and female newborns. Blood and blood-derived products were handled per applicable biohazard policies. As the type of anti-coagulant and length of storage prior to assay can affect cytokine production (31), we have established a routine standard of procedure in which blood was anti-coagulated with 15–20 U/ml pyrogen-free heparin sodium (Sagent Pharmaceuticals, Inc.; Schaumberg, IL, USA), and then kept at room temperature (RT) and processed within 4 hours (h) of collection (typically 1–2 h). Each preterm placenta was histologically examined for signs of chorioamnionitis, and information on the timing of prenatal steroid administration, as well antibiotic administration was collected. Peripheral blood was collected from healthy adult male and female volunteers employed at BCH.

**Table 1:** Characteristics of human preterm, term, and adult study participants.

	Preterm	Term	Adult
Total number of individuals	10	15	14
Site of delivery	BWH (7), BI (3)	BWH (9), BI (6)	N/A
Delivery mode	7 CS, 3 VD	15 CS	N/A
Sex	6 F, 4 M	8 F, 8 M	6 F, 8 M
(Gestational) age	28/2–34/6 weeks GA	37/0–41weeks GA	23–35 years old
Twins	1 set (Mono-Di)	1 set (pooled)	N/A
Chorioamnionitis	6 no/4 unknown	No	N/A
HIV positive status	No	No	No
Antibiotics	4 no/3 yes/3 unknown	No	N/A
Celestone	1 no/5 yes/4 unknown	N/A	N/A
Last celestone dose ≥48 h before delivery	2 no/3 yes/5 unknown	N/A	N/A

*BWH, Brigham and Women’s Hospital; BI, Beth Israel Deaconess Hospital; CS, Caesarian section; HIV, human immunodeficiency virus; VD, vaginal delivery; F, female; M, male; GA, gestational age; Mono-Di, monochorionic-diamniotic*.

### Animals

C57BL/6 mice were obtained from Charles River Laboratories and housed in specific pathogen-free conditions in the animal research facilities at BCH. To obtain newborn mice, pregnant dams were purchased on pre-determined days of pregnancy and cages checked twice daily (~every 12 h) to assess for the presence of pups. Both male and female pups were used for experiments.

### Vaccines and Whole Blood Assay

~8 ml of fresh blood was processed for the whole blood assay as previously described (32). Briefly, neonatal cord (preterm and/or term) or adult whole blood was mixed 1:1 with sterile pre-warmed (37°C) RPMI 1640 medium (Invitrogen) and 180 µl of the 1:1 suspension added to each well of a 96-well U-bottom plate (Becton Dickinson) containing 20 µl freshly prepared HBV, BCG, (BCG + HBV) at 10× final concentration, testing stimuli at a 5-point concentration–response curve based on published data (12, 33). As sources of BCG and HBV, we used the Danish Strain 1331 (Statens Serum Institut, Copenhagen, Denmark) and Recombivax^®^ HB (Merck and Co, Inc.), respectively. Suspensions containing 200 μl/well were gently mixed by pipetting and incubated at 37°C in a humidified incubator at 5% CO_2_ for 6 h.

### ELISA and Multiplex Cytokine Analysis

After treatment of the preterm, term, and adult blood with the described vaccines for 6 h, the plates were centrifuged (10 min, RT, 500 *g*), and supernatants collected and stored in three aliquots at −80°C for subsequent TNF (BD Biosciences Human TNF ELISA) and IL-1β ELISA (eBioscience Human IL-1β ELISA) and for subsequent multiplexing assays for Th1 (TNF, IL-1β, IL-12p70, IFNα, and IFNγ) and Th2 (IL-6, IL-10, and IL-12p40), and Th17 (IL-6, IL-1β) polarizing cytokines (Milliplex Human Magnetic Bead Panel; Millipore; Chicago, IL, USA). Data were analyzed on the Luminex^®^ 100/200™ System using xPOTENT^®^ software (Luminex; Austin, TX, USA).

### Isolation of Cord Blood Mononuclear Cells (CBMCs) and Peripheral Blood Mononuclear Cells (PBMCs) and *In Vitro* Stimulation

From each whole blood sample collected, matched PBMCs and CBMCs were isolated using Ficoll density gradient methodologies and cryopreserved for further downstream stimulation experiments (12, 33). MCs were stored at 50 million cells per vial in 1 ml RPMI containing 20% autologous plasma and 10% DMSO at −80°C until use. After a standardized thawing procedure, PBMCs and CBMCs isolated from human donors were resuspended at a concentration of 2 × 10^6^ cells/1000 µl of RPMI supplemented with 10% of autologous platelet-poor plasma. Cells were stimulated for 4 h with either HBV, BCG, or (BCG + HBV) (each at 1:1,000, 1:100, 1:10 vol/vol) and cells washed with ice cold PBS prior to addition of RLT buffer (RNeasy Lysis Buffer, Qiagen, MD, USA) and storage at −80°C for subsequent RNA isolation.

### Gene Expression Analysis by Quantitative Real-time PCR Array

Total RNA was extracted from lysates of vaccine-stimulated PBMCs and CBMCs using the Qiagen RNeasy Minikit and DNAse treatment performed using the Qiagen RNAase Free DNAase set all per the manufacturer’s instructions. RNA concentrations were determined using the Nanodrop 1000 and cDNA generated using the Qiagen RT2 First Strand Kit. 96-well PCR array analysis was performed using the Qiagen standardized *Innate and Adaptive Immune Reponses PCR Array* (PAHS-0522A) and RT^2^ qPCR roxSYBR green kit. Web-based PCR array analyses (RT^2^ Profiler PCR Array Data Analysis version 3.5) was used and normalized to five reference genes (B2M, HPRT1, RPL13A, GAPDH, and ACTB). Relative quantification of gene expression was calculated by the Δ*Ct* (relative expression × 10^4^). Multivariate biplots of principal component analyses were performed in R 3.4.2 using *ggplot2, ggord*, and *vegan* packages using log-fold transcript abundance of gene arrays in each group. Genes were sorted using unsupervised hierarchical heatmap clustering of log-fold changes using the *heatmap2* package.

### Immunization and Anti-Recombinant HBV Surface Antigen (rHBsAg)-Specific Ab Quantification, Subtype Classification, and Avidity Determination

For immunization experiments, mice of three age groups were used: the first group of mice were given their first immunization (prime immunization) on DOL0; the second group of mice on DOL7, and in the third group at 6–8 weeks of life. Each of the three age groups were divided into five immunization groups: saline; BCG (Organon Teknika/Merck, Durham, NC, USA) alone; HBV vaccine alone; (BCG + HBV) as a combined admixed injection; and BCG with HBV vaccine administered separately. All immunizations were injected subcutaneously (s.c.). If one injection was performed per animal, it was performed into the right posterior thigh, if two separate injections were performed they were performed either into the right and left posterior thighs (DOL7 mice; adult mice) or in DOL0 mice into the right posterior thigh (HBV) and the scruff (BCG). The injection volumes were 50 µl of vaccine (or vaccine combination)/injection in the adult animals and 25 µl of vaccine (or vaccine combination)/injection in the newborn animals (DOL0 and DOL7). The injection dose of Recombivax^®^ was 0.25 µg of rHBsAg for the adult animals and 0.125 µg in the newborn pups (DOL0 and DOL7) diluted in 0.9% NaCl Inj (USP). We chose to administer half HBV doses in the newborn as this is an established approach in human clinical vaccinology (ACIP recommendations for hepatitis B immunization). The injection dose for BCG was 0.4 × 10^6^ CFU for the adult animals and 0.2 × 10^6^ CFU for the pups (DOL0 and DOL7) diluted in 0.9% NaCL Inj (USP). We selected the BCG dose based on published literature in neonatal mice (12, 26). The selected dose of HBV was slightly lower than that routinely used in other murine studies (34) and reflected the volume limitations inherent to administration of two vaccines. We conducted preliminary experiments to confirm that we could obtain measurable Ab titers with the chosen concentration of HBV in all age groups. Mice were immunized with a prime-boost schedule; a primary immunization; and a secondary (booster) immunization, 2 weeks apart. Serum samples were obtained from blood collected *via* tail vein or artery nick as indicated for Ab detection. rHBsAg-specific IgG were quantified by ELISA. High binding flat bottom 96-well plates (Corning Life Sciences) were coated with Recombivax^®^ diluted to 1 µg/ml in carbonate buffer pH 9.6, incubated overnight at 4°C, and blocked with PBS + BSA 1% (Sigma-Aldrich) for 1 h at RT. Then, sera from immunized mice were added with an initial dilution of 1:100 and 1:3 serial dilutions in PBS + BSA 1% and incubated for 2 h at RT. Plates were then washed and incubated for 1 h at RT with HRP-conjugated anti-mouse IgG, IgG1, IgG2c (Southern Biotech). At the end of the incubation, plates were washed again and developed with tetramethylbenzidine (BD Biosciences) for 5 min, then stopped with 2N H_2_SO_4_. The optical density was read at 450 nm on a Versamax microplate reader with SoftMax Pro Version 5 (both from Molecular Devices), and endpoint titers were calculated using as cutoff two times the optical density of the background (35). For assessing Ab avidity, plates were incubated 15 min with ammonium thiocyanate 0.5 M before the addition of HRP-conjugated Abs. Avidity was expressed as the LogEC_50_ ratio of corresponding plates treated with or without ammonium thiocyanate (36).

### Statistical Analyses and Graphics

Data were analyzed and graphed using Prism for MacIntosh v. 7.0 (GraphPad Software). Tests used for statistical comparisons are indicated in figure legends. *p* value < 0.05 was considered significant. An adaptation of the Loewe method of additivity (37) was used to assess whether cytokine production after stimulation with (BCG + HBV) was synergistic, additive, or antagonistic. Concentration–response curves were subjected to regression analysis to determine the slope and *y*-intercept of each curve in the exponential phase. The formula *D* = [Ac]/[Ae] + [Bc]/[Be] was used, where [Ac] = the concentration of (HBV) used in the combination of (HBV + BCG) that results in half the maximal TNF production measured with the combination of both vaccines; [Ae] = the concentration of HBV used alone that results in half the maximal TNF production measured with the combination of (HBV + BCG); [Bc] = the concentration of BCG used in the combination of (HBV + BCG) that results in half the maximal TNF production measured with the combination of vaccines; and [Be] = the concentration of BCG used alone that results in half the maximal TNF production measured with the combination of (BCG + HBV). If *D* = 1: (HBV + BCG) act additively, if *D* > 1: (HBV + BCG) act antagonistically, and if *D* < 1: (HBV + BCG) act synergistically. Our laboratory has employed this interaction analysis method in other recently published studies (13, 38, 39).

## Results

### (BCG + HBV) Synergistically Enhances IL-1β Production in Preterm, Term, and Adult Whole Blood

**Figure 1:**
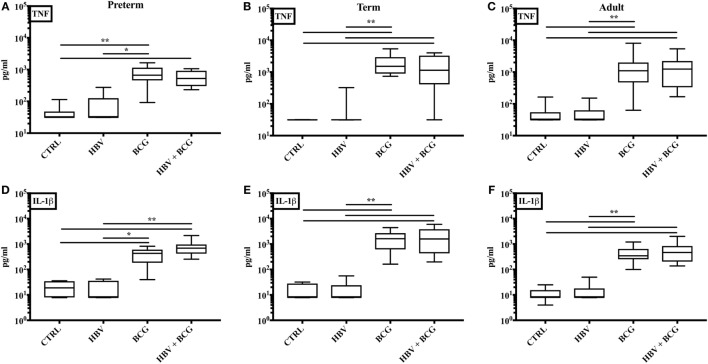
Concurrent stimulation with [Bacille Calmette–Guérin (BCG) + hepatitis B vaccine (HBV)] synergistically enhances IL-1β production in human preterm, term, and adult whole blood. Preterm **(A, D)**, term **(B, E)**, and adult **(C, F)** whole blood was stimulated with HBV alone, BCG alone, or (BCG + HBV) at 1:10 v/v. After 6 h, supernatants were analyzed for TNF **(A–C)** and IL-1β **(D–F)** cytokine production by ELISA. Statistical significance was determined by Kruskal–Wallis with Dunn *post hoc* test. *p < 0.05, **p < 0.01. *N* = 7–8 preterm, *N* = 10–11 term newborns, and *N* = 10–11 adults.

The *in vitro* whole blood assay is a useful tool to characterize the effects of vaccines on cytokine production as it enables testing of moderate number of different vaccine formulations, at multiple concentrations in a sample from a single individual (13, 38, 40). To assess whether (BCG + HBV) may enhance NF-κB and inflammasome-mediated cytokine induction compared to HBV alone, we compared (BCG + HBV) with identical concentrations of BCG and HBV in its ability to stimulate TNF and IL-1β production in preterm (Figure 1A,D) or term cord (Figure 1B,E) or adult peripheral blood (Figure 1C,F). In all three age groups, (BCG + HBV) significantly increased TNF and IL-1β secretion relative to RPMI controls and also secretion of IL-1β relative to HBV alone. Using the Berenbaum equation to assess drug–drug interactions, the interaction between HBV and BCG with regard to TNF was additive or antagonistic (Table 2). However, with respect to IL-1β, the combined vaccine effect was synergistic, defined as a *D*-value < 1, especially so in the term followed by the preterm, and least pronounced in the adult (Table 2). Of note, IL-1β is important to immunogencity of ALUM-adjuvanted vaccines (41), has a role in neutrophil recruitment and in Ab production, and has been used as an adjuvant (42–45). Figure 1 also demonstrates that BCG alone is a potent inducer of both TNF and IL-1β in whole blood of all age groups and in this context is likely the driving component behind this vaccine interaction.

**Table 2:** Quantification of (Bacille Calmette–Guérin + hepatitis B vaccine) synergism.

Age groups	*D*-value	Interpretation
**TNF**		
Preterm	13.1064	Inhibitory
Term	1.3557	Additive
Adult	1.0777	Additive
**IL-1β**		
Preterm	0.6398	Synergy
Term	0.3506	Synergy
Adult	0.7233	Synergy

### The Combination of BCG and HBV Vaccine Stimulates Secretion of Numerous Cytokines and Chemokines in Preterm, Term, and Adult Whole Blood

**Figure 2:**
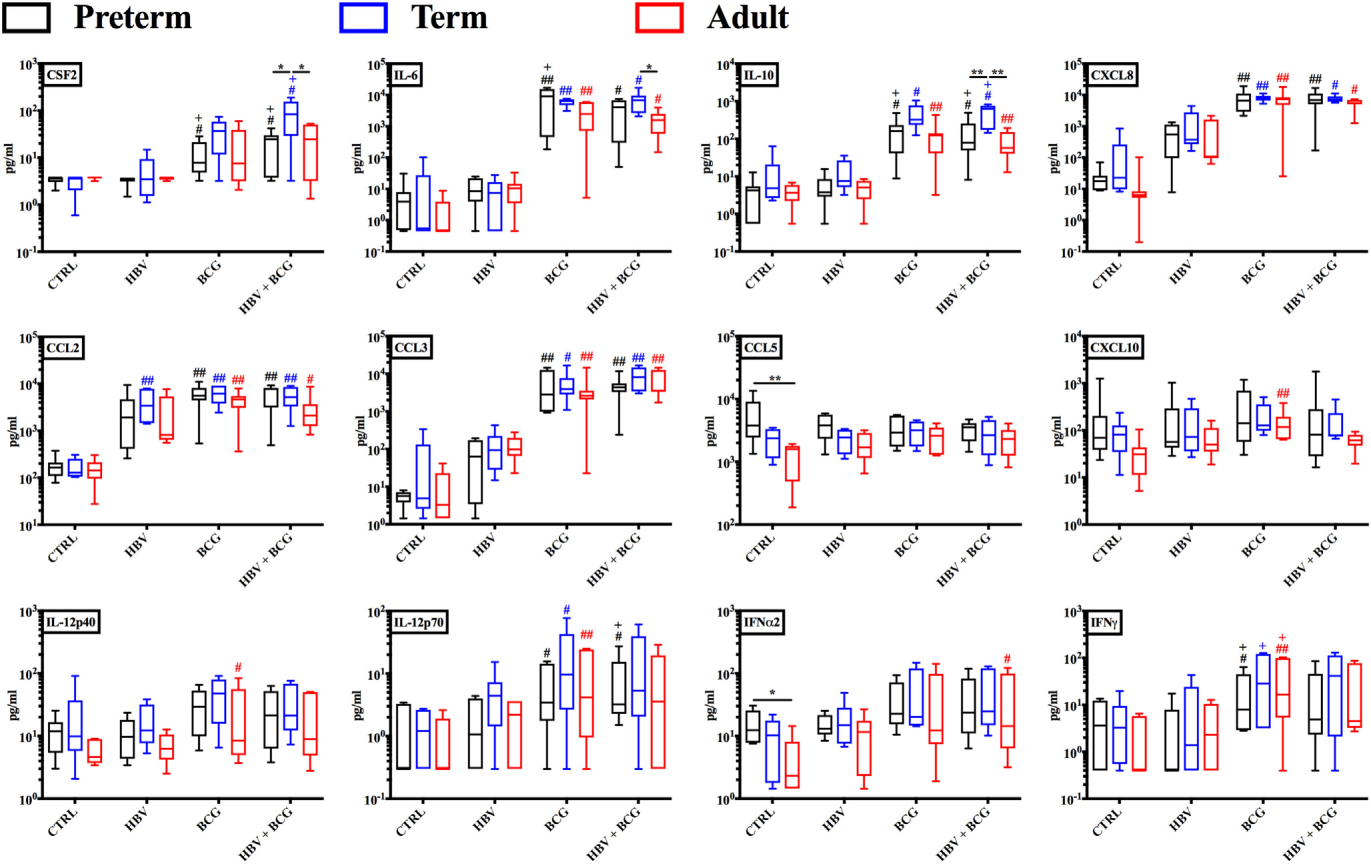
Cytokine and chemokine profiles induced by hepatitis B vaccine (HBV) and Bacille Calmette–Guérin (BCG) in human preterm, term, and adult whole blood. Preterm, term, and adult whole blood was stimulated for 6 h with either HBV, BCG, or (BCG + HBV) and supernatants analyzed *via* Multiplex Cytokine Analysis. Statistical significance was determined by repeated measure or ordinary one-way ANOVA with Holm-Sidak *post hoc* test (or their non-parametric equivalent Friedman or Kruskal–Wallis with Dunn *post hoc* test). *p < 0.05, **p < 0.01 of preterm vs. term vs. adult; ^#,+^*p* < 0.05, ^##,++^*p* < 0.01 of groups indicated by the corresponding color, respectively, vs. saline or HBV. *N* = 7 preterm newborns, *N* = 6 term newborns, *N* = 7 adults.

Having established that (BCG + HBV) enhance TNF and IL-1β production by preterm and term whole blood, we next characterized the cytokine profiles induced by these vaccine treatments in more detail employing multiplex cytokine analysis on the isolated supernatants. As shown in Figure 2, cytokines significantly induced in preterm or term cord blood by the vaccine combination relative to RPMI control and/or HBV vaccine alone included CSF2 (GM-CSF), IL-6, IL-10, CXCL8, CCL2, and CCL3. Interestingly, IL-12p70 was significantly induced by the vaccine combination in the preterm. Of note, the similarity of the BCG cytokine/chemokine profile to that induced by (BCG + HBV) suggested that the vaccine combination effect was mainly driven by BCG.

### HBV, BCG, and (BCG + HBV) Induce Distinct RNA Transcription Clusters in CBMCs/PBMCs Isolated from Preterm, Term, and Adult Individuals

**Figure 3:**
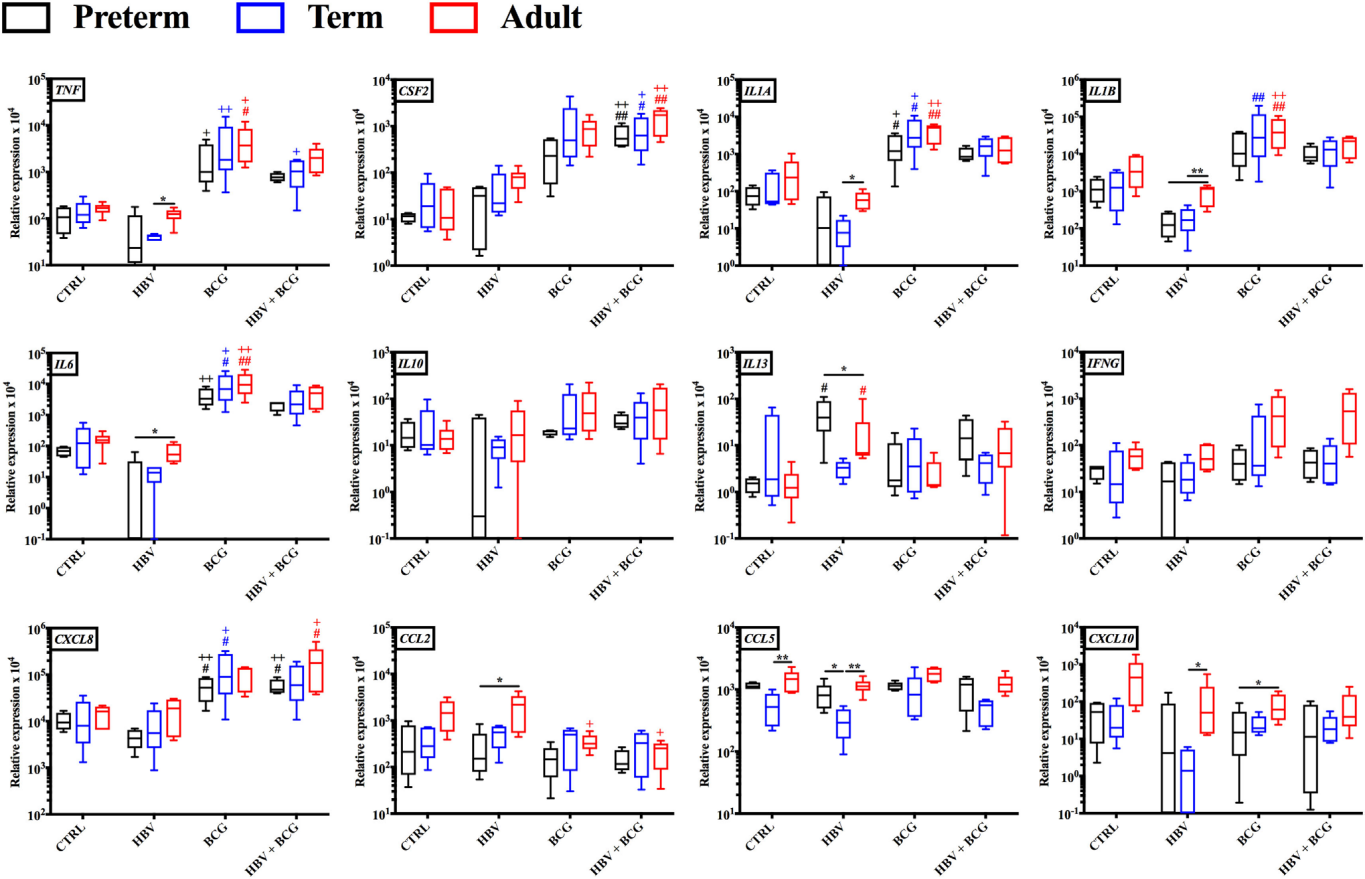
Gene expression profiles induced by hepatitis B vaccine (HBV) and Bacille Calmette–Guérin (BCG) in human preterm, term, and adult mononuclear cells. Preterm and term cord blood mononuclear cells, and adult peripheral blood mononuclear cells was stimulated for 4 h with either HBV, BCG or (BCG + HBV) and cells were harvested for quantitative real-time PCR analysis. Statistical significance was determined by repeated measure or ordinary one-way ANOVA with Holm-Sidak *post hoc* test (or their non-parametric equivalent Friedman or Kruskal–Wallis with Dunn *post hoc* test). *p < 0.05, **p < 0.01 of preterm vs. term vs. adult; ^#,+^*p* < 0.05, ^##,++^*p* < 0.01 of groups indicated by the corresponding color, respectively, vs. saline or HBV. *N* = 4–5/group.

**Figure 4:**
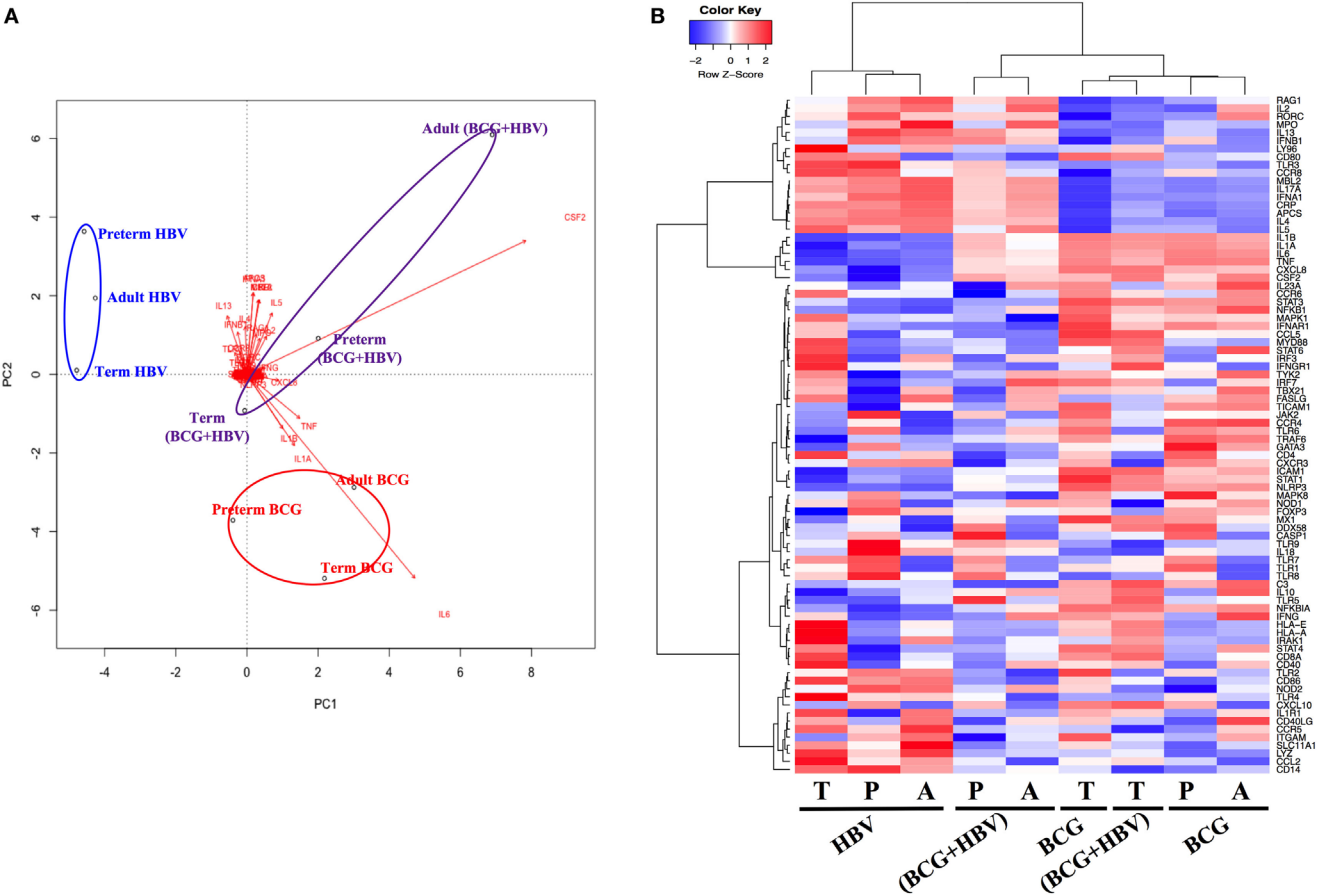
Principal component analysis of gene expression data reveals treatment-specific segregation of different age groups. Gene expression data were generated as outlined in Figure 3. **(A)** Principal components analysis biplot of mRNA gene expression data where observations (samples) are points and gene expression profiles are arrows shows dominant clustering of profiles by treatment. The distance between points approximates gene expression pattern differences among groupings. Arrows indicate genes that have greater biplot scores and drives the differences between groups. Arrowheads close to a particular group indicate genes are expressed at a greater relative abundance differences in those samples. **(B)** Unsupervised hierarchical heatmap shows clustering of treatments demonstrating log2-fold changes, expression values of genes in each sample; red to blue scale represent intensity of fold changes per genes (red indicates up, blue indicates down). Each row means individual gene and each column indicates groupings of age and treatment. P, preterm; T, term; A, adult.

To investigate how addition of BCG to HBV vaccination alters gene expression patterns at the mRNA level, RNA isolated from neonatal CBMCs (term and preterm) or adult PBMCs stimulated for 4 h with vehicle (control), BCG, HBV, or (BCG + HBV) was subjected to quantitative real-time PCR array comprised of 84 genes in human innate and adaptive immune pathways. mRNA levels were quantified in 4–5 individuals/group. Figure 3 shows that there were increases in expression of several cytokine and chemokine transcripts upon mononuclear cell stimulation with (BCG + HBV) compared to unstimulated or HBV-treated cells. Some of them reached statistical significance in all (i.e., *CSF2*) or some age groups (i.e., *CXCL8* in preterms and adults). Interestingly, some of these genes encode proteins with defined roles in vaccine efficacy (13, 42–48). Figure 4A demonstrates treatment-driven segregation of age groups by a principle component biplot of mRNA gene expression data. The points representing age and treatment group (open circles) approximate gene expression patterns between groupings. The unsupervised hierarchical heat map in Figure 4B demonstrates similar clustering by treatment and outlines in a red to blue scale high to low gene expression per gene.

### (BCG + HBV) Significantly Enhance the Level of Anti-rHBsAg-Specific IgG Titers in Neonatal Mice (Immunized on DOL0 and DOL7) but Not Adult Mice

**Figure 5:**
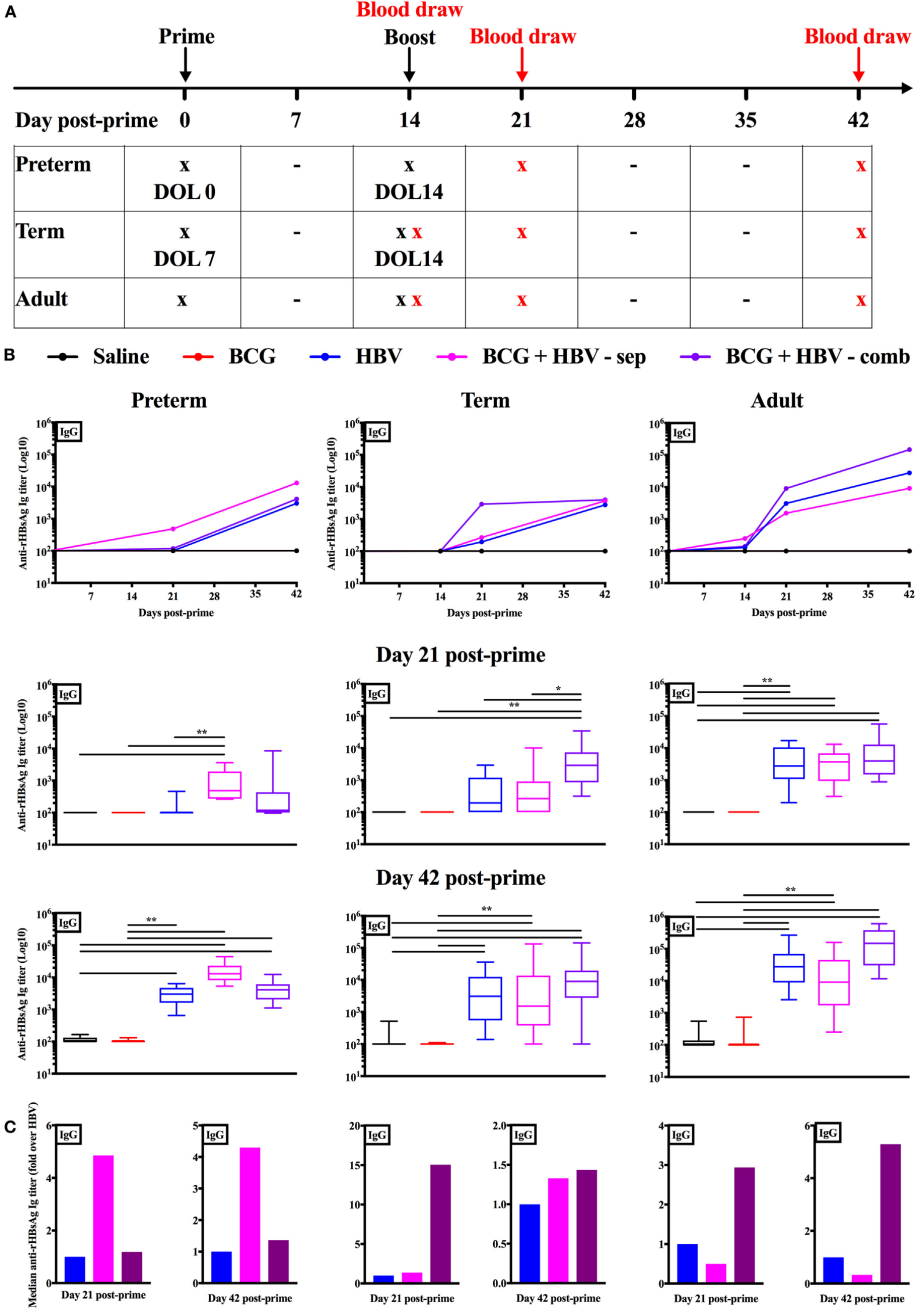
Combined immunization of newborn mice with Bacille Calmette–Guérin (BCG) and hepatitis B vaccine (HBV) *in vivo* enhances anti-recombinant HBV surface antigen (rHBsAg) IgG titers 21 days post-immunization. **(A)** Schematic representation of the immunization and blood draw schedule for the *in vivo* experiments: prime immunization was administered either on day of life (DOL) 0, DOL7, or at 6–8 weeks of life with either saline, BCG, HBV vaccine, BCG and HBV in a combined s.c. injection or BCG and HBV vaccine injected in separate sites. Booster immunization was performed with either saline in the mice prime-immunized with saline or BCG, or with HBV vaccine in the mice prime-immunized with either HBV vaccine alone, or BCG and HBV (either in combined or separate injection). Blood draws in the adult mice were obtained prior to prime immunization, prior to booster immunization, at 21 days and at 42 days post-prime immunization. Blood draws in the neonatal mice immunized on DOL7 were performed prior to booster immunization, at 21 days and at 42 days post-prime immunization. Blood draws in the mice immunized on DOL0 were obtained at 21 days and at 42 days post-prime immunization. Some neonatal mice from both the group immunized on DOL0 as well as DOL7 were sacrificed for a baseline blood draw prior to prime immunization. **(B)** Anti-rHBsAg IgG titers in mice immunized on DOL0, mice immunized on DOL7 and at 6–8 weeks of life. **(C)** Fold change over the HBV vaccine immunized group of the median anti-rHBsAg IgG titers. *N* = 9–14/group for the mice immunized on DOL0; *N* = 14–16/group for the mice immunized on DOL7; *N* = 15–17/group for the mice immunized at 6–8 weeks of life. Data are representative of two independent experiments each of which included all three age groups and within each age group all five treatment groups. Statistical analysis of differences between the treatment groups was performed *via* Kruskal–Wallis test with Dunn’s *post hoc* test. *p < 0.05, **p < 0.01.

To assess the impact of (BCG + HBV) *in vivo*, we turned to a murine model. Mice at DOL 0–5 have been utilized as a model for preterm innate and adaptive immunity (49, 50) while mice at DOL7 have been used to model the term newborn (4, 15, 51–53). We made use of these age-specific models to investigate whether BCG can enhance early-life immunization with HBV, for which anti-Hepatitis B sAg Ab titers are the established correlate of protection (54). Figure 5A demonstrates the schedule according to which the mice of all three age groups were prime-immunized and booster immunized 2 weeks later. The age at prime immunization was DOL0 (the “preterm” group, *n* = 9–14), DOL7 (the “term” group, *n* = 14–16) and 6–8 weeks for the adult group (*n* = 15–17). These data were acquired in two separate experiments, each of which included all three age groups and within each age group all five treatment groups. The linear graphs represent median anti-rHBsAg IgG titers over time post-prime immunization (Figure 5B). The box-and-whisker plots depict Ab titers 21 and 42 days post-prime immunization. To assess whether the potential beneficial effect of addition of BCG to HBV depends on co-administration of both vaccines into the same site, we differentiated two combined treatment groups: one in which (BCG + HBV) were combined (i.e., admixed) and injected into the right flank s.c. and one in which HBV was injected into the right thigh and BCG was injected either into the scruff (neonatal mice on DOL0) or left thigh (neonatal mice on DOL7 and adult mice). Addition of BCG to HBV significantly enhanced Ab responses at 21 days post-prime immunization in neonatal mice immunized on DOL0 and DOL7 but not in adult mice. Whereas in DOL0 mice, it was the separate injection of BCG and HBV that significantly enhanced anti-rHBsAg IgG titers, the combined injection was the administration technique that lead to enhanced Ab titers in DOL7 mice. While this effect was sustained at D42 post-prime immunization in mice immunized on DOL0, it was no longer evident in neonatal mice prime-immunized on DOL7 (Figure 5C). Interestingly, switching toward IgG2c was observed only in adult mice immunized with (BCG + HBV) (Figure S1 in Supplementary Material). In addition, BCG did not significantly modulate Ab avidity, suggesting that BCG did not affect affinity maturation of anti-rHBsAg Abs (Figure S2 in Supplementary Material).

## Discussion

In this study, we have demonstrated for the first time that BCG, alone or when coadministered with HBV in a neonatal context, can enhance human innate cytokine responses *in vitro*. Moreover, we show that BCG can enhance hepatitis B antigen-specific murine adaptive responses *in vivo*. These observations are important in that newborns and young infants are highly susceptible to infection with intracellular pathogens including viruses, such as hepatitis B virus. Acquisition of hepatitis B virus during the newborn period carries risks of developing both hepatocellular carcinoma and liver cirrhosis. Moreover, most licensed vaccines, including HBV, are not optimally effective at birth and require multiple booster immunizations later in life despite the fact that hepatitis B immunization in newborns induces higher primary and memory Ab responses than in adults (55). Under the current immunization schedule with an ALUM adjuvanted HBV vaccine, the term newborn does not reach a status of immunological protection against HBV, as measured by titers of anti-rHBsAg Abs, until 6 months of life. For the preterm newborn with a birth weight <2 kg the unmet need to enhance responses to HBV is exacerbated by the fact that a birth dose is not recommended as priming has been inefficient in this population. With age-specific differences in the quantity and quality of cellular and soluble factors playing a role (56), the neonatal immune system is distinct from that of infants and adults, with bias toward induction of regulatory T cell and Th2-type T cell responses. Distinct early-life immunity limits the efficacy of adjuvants that activate newborn DCs to produce Th1-polarizing cytokines. The preterm newborn, in addition to demonstrating low innate Th1 support, demonstrates a low TLR-mediated production of Th17-supporting cytokines, with robust anti-inflammatory IL-10 levels (57–60). Combined stimulation of newborn cells through certain combinations of PRRs may potentially overcome the early-life bias against Th1 responses (30, 38, 61). In summary, there is an unmet need for early-life vaccine strategies that provide earlier protection against HBV infection for the term and in particular for the preterm infant.

While HBV immunization of the term infant effectively reduces chronic Hepatitis B infection in countries with universal neonatal immunization, the window of vulnerability in term and preterm infants at birth (into infancy) is still a major concern. This concern could be addressed by maternal or an optimized (one dose) HBV immunization of the newborn at birth. The common goal among maternal vaccination programs is temporary protection of the young infant against severe illness and death by ensuring sufficient and timely transfer of protective antibodies from the mother (62). Unfortunately, while some attempt have been made, clinical studies of vaccination against hepatitis B have showed lower immunogenicity in pregnant women than in nonpregnant women (63, 64). Regarding improved immunization of the newborn, as the Center for Disease Control outlines, worldwide, most people with chronic Hepatitis B are infected at birth or during early childhood. While a positive mother is a significant source of risk for Hepatitis B infection as a newborn, the recommendation to administer Hepatitis B vaccination at birth in many countries including the United States is based on the understanding that asymptomatic chronic carriers within the family and family contacts can be a significant risk for infection in the neonatal period (ACIP, recommendations for Hepatitis B immunization).

BCG is the most commonly administered vaccine in world history with billions of doses given globally at or soon after birth to protect against disseminated TB during infancy (65). In addition to its protective effects against TB, for which there is no established correlate of protection, BCG administration may also enhance Ab responses to unrelated pathogens in human newborns and infants (20, 27, 28) and in newborn mice (26). Protective heterologous effects of certain live-attenuated vaccines including BCG have been demonstrated that reduce morbidity and mortality beyond what can be attributable to prevention of the target disease (23, 25). BCG activates TLRs, including TLR2 and TLR4 (66), as well as C-type lectin receptors (CLRs) such as Dectin-1 and macrophage-inducible C-type lectin (Mincle) (67–70), resulting in strong Th1-biased immune responses. Of note, combined TLR and CLR activation synergistically enhances Th1- and Th17-cytokine induction in human newborn monocyte-derived dendritic cells (38), raising the possibility that engagement of multiple PRRs by BCG may contribute to the observed enhancement of HBV immunogenicity. Accordingly, we examined the cytokine profiles induced by (BCG + HBV) relative to HBV alone in term and moderate to late preterm human infants on the protein and mRNA levels *in vitro* and then to examine the impact of cytokine polarization on the adaptive anti-rHBsAg-specific Ab production *in vivo* utilizing both mice on DOL0 as well as DOL7 to mirror different levels of immune ontogeny.

Our study characterized (BCG + HBV)-induced human leukocyte cytokine profiles at different GAs at the protein and mRNA level. Of these *IL1B, IL*6, *CSF2*, and *TNF* were key drivers of gene clustering in the principal component analysis by treatment instead of by age. The interleukins induced are particularly noteworthy as they have been associated with enhanced adaptive immunity: (a) IL-1β, whose production was synergistically induced by (BCG + HBV) in human preterm and term cord blood as well as peripheral adult blood *in vitro*, is an inflammasome-produced cytokine that may improve vaccine immunogenicity (42–45), having a role in neutrophil recruitment (71) and in Ab production (72) and has been used directly as an adjuvant (73). Indeed, IL-1β has been implicated as important to immunogenicity of HBV, in particular anti-HBsAg Ab responses (74–76) and (b) IL-6, a Th17-polarizing cytokine that stimulates differentiation and maturation of B cells to Ab-producing plasma cells, stimulates T cell proliferation, and is a murine adjuvant (46, 47, 77).

In neonatal mice, compared to HBV alone, we found that (BCG + HBV) induced significantly higher anti-rHBsAg-specific IgG levels at 21 days after prime immunization. In DOL0 mice, this effect was significant when BCG and HBV were injected into separate sites, in DOL7 mice this effect was significant when both vaccines were injected in combination. Although it should be recognized that the kinetics of immune ontogeny are distinct in mice compared to humans, this relatively early effect of BCG co-administration is intriguing. If such enhancing effects of BCG would extend to humans, they may fall within the window of susceptibility inherent to current term infant immunization schedules, prior to completion of HBV booster immunization. While in adult mice, the (BCG + HBV) group demonstrated higher Ab titers, the difference relative to HBV alone did not reach statistical significance. A surprising finding of this study was the observation that in the DOL0 mouse the separate injection of BCG and HBV compared to a combined injection in the same age group was the potent route of administration whereas in both the DOL7 mouse as well as in the adult mouse, the combined injection was more effective than the separate administration. This observation could reflect age-specific immunity and deserves further investigation. In summary, our study demonstrates that (BCG + HBV) synergistically induced IL-1β *in vitro* and enhanced neonatal anti-rHBsAg-specific Ab titers at an early stage post prime and first booster immunization *in vivo*. Given evidence in mice and humans that IL-1β production enhances the magnitude of HBV-induced Ab responses (74–76), we speculate that robust (BCG + HBV)-induced inflammasome activation may contribute to the observed enhancement in HBV immunogenicity.

Our study features many strengths including, to our knowledge, multiple novel aspects: (a) an age-specific approach to characterizing vaccine–vaccine interactions including study of preterm humans and newborn DOL0 mice, reflecting aspects of preterm humans who represent ~11% of all global live births and are particularly susceptible to infection (3); (b) human *in vitro* modeling of (BCG + HBV) effects, in a way that reflects immune ontological differences present in these age groups *in vivo*; (c) evaluating the impact of vaccine–vaccine interactions on innate cytokine induction using mathematical and bioinformatic approaches, and (d) characterizing age- and administration- (e.g., combined vs. separate injections) specific BCG-HBV interactions in newborn mice *in vivo*.

Our study also has a number of limitations. With regard to our *in vitro* systems, although providing potentially valuable human data that have predicted adjuvantation effects *in vivo* (13), they may not optimally reflect vaccine effects *in vivo*. The use of a whole blood assay aims to reflect *in vivo* conditions including differences in cell quantity and immunphenotype between the neonate and the adult. Consequently, whole blood data are limited with regard to the ability to ascribe cytokine differences to single cell function or cell composition (78). Moreover, there are differences in the functionality and composition of mononuclear cells from adult individuals and mononuclear cells derived from neonatal cord blood (e.g., more predominantly lymphocytic) (78). Potential confounders in the use of preterm cord blood include maternal disease such as preeclampsia, a disease not captured in our collection of data for the preterm cord blood samples. We were able to limit the effects of steroid exposure on our sample collection for a group of preterm cord blood samples as steroids were known to have been given over 48 h prior to cord blood collection (Table 1). We also recognize that attention must by paid to the type of anti-coagulant used for peripheral or cord blood collections as the type of cytokine used can affect cytokine production. We chose pyrogen-free anti-coagulant heparin sodium because it is certified to be endotoxin-free. Future studies may need to compare the results obtained with other methods of anti-coagulation, such as EDTA. Our *in vivo* studies feature distinct species (mouse) and route of administration (subcutaneous) from human newborns (intradermal). Indeed, we were not able to demonstrate higher primary anti-HBV Ab responses in our neonatal mice relative to our adult mice as has been previously demonstrated in humans (55). Nevertheless, the cogent pattern of enhanced age-dependent HBV responses in the presence of BCG, mirroring those observed in some clinical cohorts (20) suggests that our data may be relevant to the effects of these vaccines in human newborns *in vivo*.

Future work will be necessary to elucidate the immunological mechanisms involved in the BCG adjuvantation phenomenon described here, and hence enable design of a new generation of vaccines that recapitulate desirable features of the live vaccine BCG as (a) a single dose effectiveness and (b) induction of both adaptive and trained immunity. At this point, it is unclear whether the observed BCG-driven phenomena relate mechanistically to “heterologous” effects that could be mediated by trained immunity (79). In addition to informing optimization of the use of BCG vaccine together with other vaccines, characterizing BCG-induced enhancement of Ab titers in response to unrelated vaccines may inform development of “BCG-like” adjuvantation systems (12). Furthermore, of importance to global health, these findings support the hypothesis, that in the appropriate context in countries in which neonatal immunization with BCG is recommended, concurrent administration of (BCG + HBV) at birth to the moderate to late preterm and term newborn may enhance the protective response to HBV immunization. Of note, in relatively small preterm studies thus far, BCG has been immunogenic and safe when administered to the moderately to late preterm infant [31–33 weeks GA] (80). Further studies of the safety, efficacy and mechanism of action of the combination of (BCG + HBV) compared to each alone in newborn animals, including humans, will shed further light into this important area crucial to the protection of the most vulnerable among us.

## Ethics Statement

Local institutional review boards at The Brigham and Women’s Hospital (Protocol #2000P000117/BWH) and the Beth Israel Deaconess Medical Center (Protocol #2011P-000118/BIDMC) have approved the cord blood collection protocols. Patient information concerning the collected cord blood samples was collected in a de-identified manner as approved by the onsite IRB. Peripheral blood draws were conducted at Boston Children’s Hospital (BCH) from healthy adult volunteers, employed at BCH after written informed consent with approval from the Ethics Committee of BCH (protocol number X07-05-0223). All experiments involving animals were approved by the Animal Care and Use Committee of BCH and Harvard Medical School (protocol numbers 15-11-3011 and 16-02-3130).

## Author Contributions

AS, FB, DD, and OL designed the study. AS, DD, IB, and HC collected cord blood samples. AS, DD, MP, SJ, NL, and IB conducted the *in vitro* experiments. AS, FB, and CP conducted the *in vivo* experiments. JD-A performed the RNA data analysis. TK shared knowledge in the design of whole blood assays. AS and FB wrote the manuscript. DD and OL provided overall mentorship and assisted in writing and editing the manuscript. FB, CP, JD-A, MP, HC, SJ, NL, IB, and TK contributed to helpful discussions and review of the final manuscript. All the authors have given final approval for the version submitted for publication.

## Conflict of Interest Statement

The authors declare that the research was conducted in the absence of any commercial or financial relationships that could be construed as a potential conflict of interest. The Levy Lab has received past sponsored research support from MedImmune, Crucell (Johnson & Johnson), and 3M Drug Delivery Systems that is not directly relevant to the content of this manuscript.
