# Supplementary material for: Adjuvant Effect of Bacille Calmette–Guérin on Hepatitis B Vaccine Immunogenicity in the Preterm and Term Newborn
Source: Front Immunol. 2018 Jan 24;9:29. doi: 10.3389/fimmu.2018.00029 (PMC5787546; doi:10.3389/fimmu.2018.00029)
Supplement: Supplementary file 1 [file Image_1.PDF]

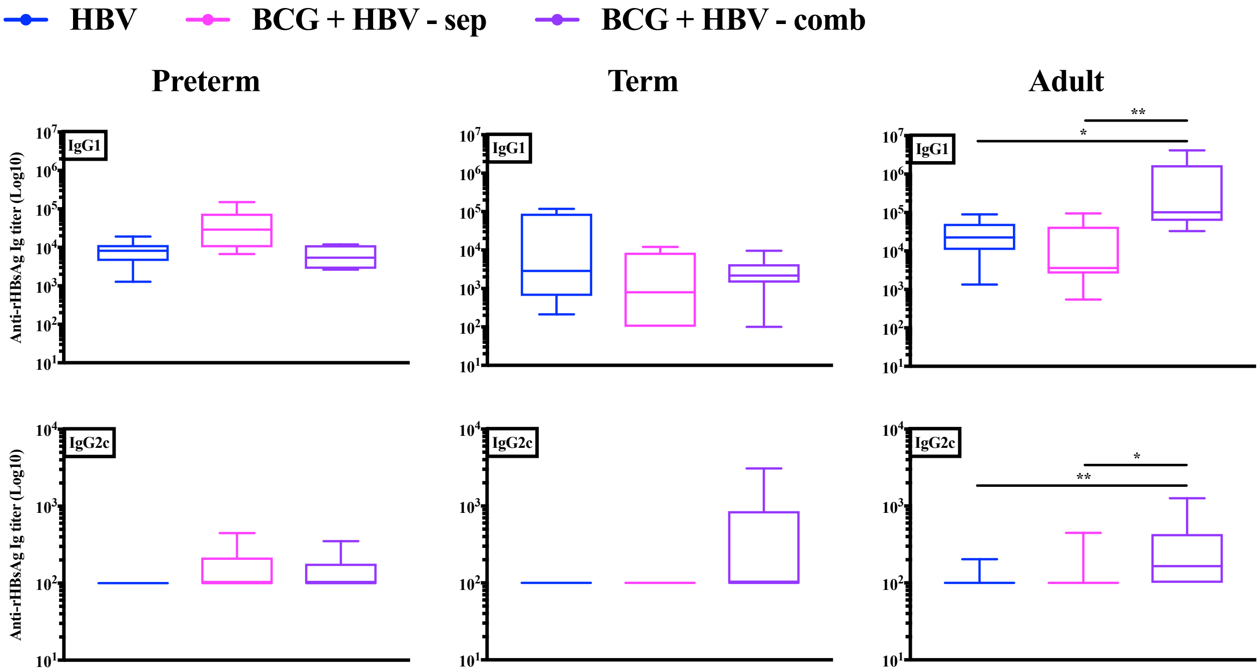

Supplementary Figure 1

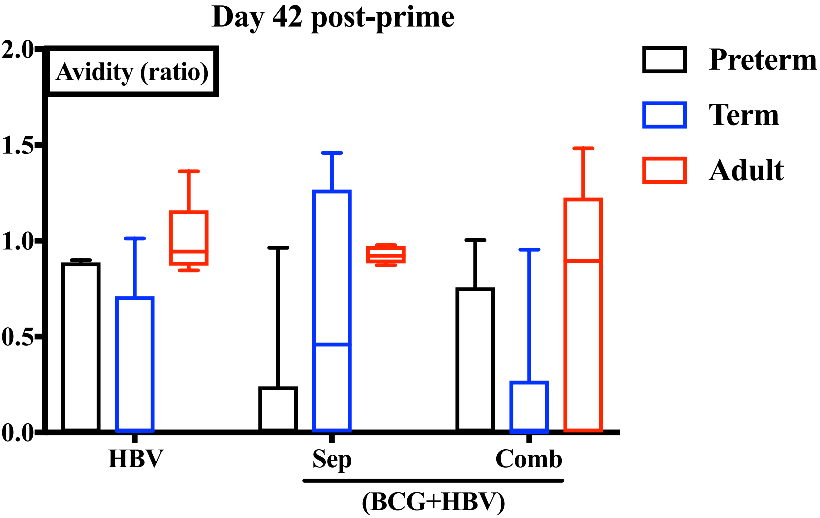

Supplementary Figure 2

Supplementary Figure 1. Preterm, term and adult mice were immunized with HBV alone or with BCG (in a combined or separate injection) and serum samples were collected as indicated in Figure 4A. Anti-rHBsAg IgG1 and IgG2c titers were determined by ELISA at day 42 post-prime. Statistical significance was determined by Kruskal-Wallis test with Dunn's post hoc test. *$p < 0.05$, **$p < 0.01$. N = 6-8 mice for the preterm and term groups; N = 11-14 mice for the adult group.

Supplementary Figure 2. Preterm, term and adult mice were immunized with HBV alone or with BCG (in a combined or separate injection) and serum samples were collected as indicated in Figure 4A. Avidity of anti-rHBsAg IgG was measured by ELISA and expressed as the ratio between the LogEC50 values obtained with and without ammonium thiocyanate treatment (0.5 M). Statistical significance was determined by ordinary one-way ANOVA with Holm-Sidak post hoc test (or its non-parametric equivalent Kruskal-Wallis with Dunn post hoc test). N = 5-6 mice per group.
